# Are the Organoid Models an Invaluable Contribution to ZIKA Virus Research?

**DOI:** 10.3390/pathogens10101233

**Published:** 2021-09-24

**Authors:** Pasquale Marrazzo, Monica Cricca, Claudia Nastasi

**Affiliations:** 1 Department of Experimental, Diagnostic and Specialty Medicine, Alma Mater Studiorum University of Bologna, 40126 Bologna, Italy; 2 Section of Microbiology, Department of Clinical and Experimental Medicine, University of Bologna, 40138 Bologna, Italy; monica.cricca3@unibo.it; 3 Center for Applied Biomedical Research, S.Orsola-Malpighi University Hospital, 40138 Bologna, Italy; 4 Department of Oncology, Laboratory of Cancer Pharmacology, Istituto di Ricerche Farmacologiche Mario Negri IRCCS, 20156 Milan, Italy

**Keywords:** organoids, cellular microbiology, organotypic, 3D cell model, tissue engineering models, in vitro models, viral research, Zika virus

## Abstract

In order to prevent new pathogen outbreaks and avoid possible new global health threats, it is important to study the mechanisms of microbial pathogenesis, screen new antiviral agents and test new vaccines using the best methods. In the last decade, organoids have provided a groundbreaking opportunity for modeling pathogen infections in human brains, including Zika virus (ZIKV) infection. ZIKV is a member of the Flavivirus genus, and it is recognized as an emerging infectious agent and a serious threat to global health. Organoids are 3D complex cellular models that offer an in-scale organ that is physiologically alike to the original one, useful for exploring the mechanisms behind pathogens infection; additionally, organoids integrate data generated in vitro with traditional tools and often support those obtained in vivo with animal model. In this mini-review the value of organoids for ZIKV research is examined and sustained by the most recent literature. Within a 3D viewpoint, tissue engineered models are proposed as future biological systems to help in deciphering pathogenic processes and evaluate preventive and therapeutic strategies against ZIKV. The next steps in this field constitute a challenge that may protect people and future generations from severe brain defects.

## 1. General Introduction

The zika virus (ZIKV) is a vector borne-flavivirus, with a single serotype, but two differentiated lineages: the East/West African and the Asian genotypes [1].

ZIKV is closely related to several other pathogens that cause disease globally, including Dengue (DENV), yellow fever (YFV), West Nile (WNV), Japanese encephalitis (JEV), and tick-borne encephalitis (TBEV) viruses [2]. Among mosquitos, the genus *Aedes* is capable of carrying and transferring ZIKV to humans, with *Aedes aegypti* and *Aedes albopictus* being the main vectors as they feed almost exclusively on humans. Although most ZIKV infections are transmitted by infected mosquitoes, ZIKV transmission can occur through sexual intercourse contact, blood transfusion, laboratory exposure, and both intrauterine and intrapartum transmission. The viral transmission via breastfeeding has not been reported.

ZIKV was first isolated in sentinel rhesus macaque monkeys in 1947 in Uganda and later in humans in 1952 [3]. For a long period, it was not considered a threat due to the mild and self-limiting symptoms following the infection of most people. Later on, in 2007, the virus spread outside the African territory and caused the first major outbreak on the Micronesian island of Yap where 70% of the inhabitants got infected. Another major outbreak occurred in 2013 in French Polynesia and within two years, in March 2015, it reached the Americas when Brazil reported a large outbreak of rash illness. In July of the same year, ZIKV was associated with Guillain–Barré syndrome in adults; then in October, the first link of microencephaly in newborns of infected mothers was reported. This association was supported also by in vivo studies where the virus infected neural progenitors and impaired the brain development [4]. These epidemics led the World Health Organization to declare ZIKV disease as a public health emergency in February 2016 and then to withdraw the claim on November 2016, redefining it as serious public threat but not an emergency [5].

In general, the most relevant feature of ZIKV with respect to other of Flaviviridae family relies on its ability to target the nervous system and induce the onset of neurological symptoms in fetuses and infants.

Infections during pregnancy are critical for fetuses, especially when intrauterine transmission occurs during the first trimester and the immature neurons are susceptible to ZIKV [6]. In addition, the virus is able to cross the placental barrier by targeting placental macrophages [7] and, due to the neurotropism, to attack the fetus causing brain damage for the newborn [8]. In addition to congenital disease, ZIKV can cause visceral [9] and other neurotropic diseases such as meningitidis in adults. Usually, when a symptomatic infection occurs, is mostly mild, such as with fever, rash, myalgia, arthralgia. The incubation lasts between 4 and 13 days and those symptoms do not usually last longer than a week. In some cases, other complications may arise, such as Guillain–Barré syndrome with poor prognosis.

Different in vitro and in vivo models confirmed that the neural progenitor’s infection in the mammalian brain constitutes the pathogenetic key leading to the hypomorphic effect, but more complex experimental models are needed to address subcellular pathogenesis events, allowing the trials of new therapeutic interventions.

Up to date, a total of 86 countries and territories have reported evidence of mosquito-transmitted Zika infection and the current epidemic is caused by ZIKV from an Asian lineage [10] and there is no vaccine or approved small-molecule based drug to prevent or efficiently treat ZIKV infection.

It is well-known that Zika spreads similar to other arboviruses [11] and, as ZIKV and Dengue (DENV) viruses share the same arthropod vector, the extent and effects of the immunologic cross-reactivity are of great interest for the scientific community [12]. This relatedness raises the potential of Antibody Dependent Enhancement (ADE) via cross reactive antibodies to West Nile Virus (WNV) and Dengue [13]. Given the actual limitations on vaccine design against ZIKV infection, passive vaccination by using monoclonal neutralizing antibodies (MAbs) is consequently one of the most studied strategies for treating the infection. However, most of MAbs tested to date have shown a weak response against ZIKV, which, again, could create the risk of developing ADE related to ZIKV infection [14,15].

## 2. ZIKV Features

Zika virion has a diameter of 50 nm with a 11 kb positive-stranded RNA molecule that encodes three structural and seven non-structural proteins. The structural proteins: capsid (C), membrane (prM/M) and envelope (E) aim at assembling the viral particle, whereas the other seven are non-structural proteins (NS1, NS2A, NS2B, NS3, NS4A, NS4B and NS5) are used by the virus to orchestrate viral replication, avoid host defenses and exploit the host replication machinery. Furthermore, while the sequences of ZIKV NS3 and NS5 proteins are highly similar to other flaviviruses, the sequences of prM, E and NS3 are significantly different [16]. For this reason and due to its abundance, the protein E (which consists of three ectodomain DI, II, III) is considered to be the most important, and has a great relevance in the search for vaccines or neutralizing antibodies against ZIKV infection [17,18].

The ZIKV life cycle depends on the cellular secretory pathway for complete virion formation, maturation and release. After the mosquito bite, ZIKV infects nearby skin cells, and then it is delivered to the draining lymph nodes after being picked up by skin-resident dendritic cells. In the lymph nodes, ZIKV infects monocytes and macrophages and then disseminates to other body sites, including placenta and testes.

Translation occurs within the ER membranes. The nascent virions accumulate within the ER lumen and are delivered to the next secretory compartment, the Golgi complex. Immature virions accumulate in the ER-Golgi hybrid compartment where the viral surface glycoproteins undergo final glycosylation and maturation. Virions can be secreted by a secretory autophagy-related pathway as membrane-wrapped clusters of virions (pathway 1), alternatively virion clusters could be released via direct fusion of the membranous compartments with the plasma membrane (pathway 2). However, the major mechanism of virion egress is via a secretory granule-like mechanism as individual virions (pathway 3) [19].

Extracellular vesicles (EVs) represent an important mode of intercellular communication between cells by delivering cytosolic proteins, lipids, and RNA. In the same extent of hepatitis C virus (HCV), it has been recently reported that Zika virus also hijacks EVs pathways to package viral components that are infectious and potentially less immunogenic than mature virions. As EVs have been shown to cross blood–brain and placental barriers, it is possible that Zika virus could gain access to the brain or developing fetus via this mechanism. In particular, Zika virus-infected cells secrete distinct EV subpopulations with specific viral protein profiles and infectious genomes with respect to those released from non-infected cells [20].

It is known that most pantropic viruses employ similar dissemination strategies. At the inoculation site virions replicate in tissue’s macrophages and dendritic cells, which traffic the virus to the draining lymph nodes and other lymphoid tissues. In these latter, large numbers of macrophages are recruited and amplify the virus spreads to monocytes, macrophages or dendritic cells in multiple tissues reaching the remaining tissues. In fact, in rhesus macaques, in situ hybridizations have demonstrated that neurons, macrophages, neutrophils, and dendritic cells are permissive [21,22,23]. Not only it can persist in the macaque central nervous system but also in the lymphoid tissue for over 4 weeks after infections [23,24].

Indeed, ZIKV RNAs can be found in many biological fluids, so it can be diagnosed by detecting its RNA in blood, urine, and other body fluids by reverse-transcriptase–polymerase-chain-reaction (RT-PCR) assay. However, the length of time that it remains detectable in such fluids is not clear. ZIKV can also be diagnosed by assessing the presence of anti–ZIKV IgM antibodies in the blood. [25].

## 3. ZIKV in Vertical Transmission and Congenital Diseases 

The placenta is the main organ appointed to filter toxic molecules, allowing the exchange of nutrients and solutes and acting as a specialized interface between the mother and the developing fetus [26], as well is fundamental for pathogen’s vertical transmission. In addition, it naturally acts as an immune barrier that enables the active transport of maternal immunoglobulins G (IgG) by neonatal Fc receptors to the fetus [27]. However, many viruses such as rubella virus, cytomegalovirus and herpes simplex viruses can cross the placenta by-passing its immune protection. ZIKV is a TORCH group member (*Toxoplasma gondii*, other, rubella virus, cytomegalovirus, herpes simplex virus, parvovirus B19) [28], an acronym coined referring to several pathogenic viruses, bacteria and parasites known to traverse the maternal–fetal barrier, causing miscarriage and congenital anomalies [27]. The placentae show histopathological damage and abnormal organelles at an ultrastructural level during ZIKV infections [26].

The sequelae of infections in pregnancy usually include teratogenic effects, that cause congenital anomalies which the most common are ventriculomegaly, lissencephaly, hydranencephaly, microcephaly, developmental delays [29] and ocular abnormalities [28]. Regrettably, the state-of-the-art about other flaviviruses highlight the multifactorial complexity of ZIKV vertical transmission. The mechanisms by which ZIKV crosses the placenta causing defects and subsequent adverse birth outcomes are yet to be fully discovered [30] but studying it through the developmental stages of placenta may help to understand it. In detail, its development is not fully complete until the end of the first trimester as the chorionic villi are still forming; once the uteroplacental circulatory system is established, the placenta represents the unique barrier preventing pathogens to access. Studies carried on human placental explants and mouse models suggest differences in transmission efficacy and fetal sequelae at different gestational stages, with the first trimester being at the greatest risk. Decidual cells have been suggested to be the primary site of ZIKV replication [31], thus being a critical TORCH reservoir at the maternal–fetal interface. As the virus can get through adjacent target cells, from the decidua basalis to the anchoring villi, ZIKV tropism during the first trimester of pregnancy can partially explain the congenital damages [32]. For example, while the VP2 capsid protein of parvovirus B19 has been shown to bind syncytiotrophoblast (STB) at placenta level, TAM receptor expression associated to decidual cells are not essential for in vivo mouse ZIKV infection [33]. In several animal models was shown that ZIKV infects trophoblasts and fetal endothelial cells thus that its RNA was detected in the fetal brain and in the placenta of spontaneously aborted human fetuses during the first and second trimesters [34]. Then, gestational stage and many host genetic factors may affect the relative vulnerability in vertical transmission of ZIKV [35]. Neonatal PCR testing, placental findings, and infant outcomes can be discordant between co-twins with antenatal ZIKV exposure demonstrating that each twin should be evaluated independently for vertical transmission [36]. In general, studying TORCH pathogens’ vertical transmission is limited by the poor availability of both human clinical samples and primary tissues. On the other hand, the development of organoid of both maternal and fetal cells introduced new avenues to study the maternal–fetal interface, but mimicking its immunological complexity is still a challenge.

Similar to ZIKV, neonatal herpes-simplex-virus and congenital-cytomegalovirus infections are associated with neurological deficits such as microcephaly [37]. In fact, many are the viruses that affect the central nervous system (CNS) at distinct stages of life [38]; as such, neurotropic RNA viruses (e.g., rabies, Japanese and Eastern Equine Encephalitis, Ebola, West Nile, Powassan) infect the brain and spinal cord, causing meningitis, encephalitis, microcephaly, and Guillain–Barré syndrome [39]. In general, efforts are expected to add value to current organoid technology to better explore the complexities of neurotropic viruses within the CNS; recently, in fact, brain organoids were used for modelling the neurotropic effects of SARS-CoV-2 and comprehensively understanding the neurological effects of the infection [40].

## 4. ZIKV and the Host Immune Responses

The knowledge of viral entry receptors and cellular tropism is fundamental for understanding the mechanisms of viral immunity and pathogenesis.

While the E protein has been appointed as the viral receptor for the binding and fusion, several are the candidate molecules as entry receptors, which many are shared with dengue virus (DENV).

In clinical samples putative target receptors have been identified in glial cells, amniotic epithelial cells, fetal mesenchymal cells, neural progenitors’ cells, glial cells, and the macrophages of the placenta (Hofbauer cells) [34,35,41,42]. Depending on the cell type the entry receptors may vary from C-type lectins, mannose receptors, dendritic cell-specific intercellular adhesion molecule-3-grabbing non-integrin (DC-SIGN or CD209) for dendritic cells, or the phosphatidylserine receptors T cell immunoglobulin and mucin domain (TIM) and Tyro3, Axl, Mertk (TAM). Although one TAM family member was previously involved in ZIKV entry in cell lines and primary human cells, more recent studies in primary human neural progenitor cells and brain organoid cultures [43] have shown no effect of either Axl or Mertk on ZIKV infection [44,45,46].

A short course of illness and self-limiting febrile symptoms in most cases of *Flavivirus* infections implicate a role of the innate immune system in controlling their infections. Usually, the type I interferons (IFN-α, β) system is triggered within hours of viral infection upon recognition of the virus by pattern recognition receptors (PRRs) such as retinoic acid-inducible gene I (RIG-I)-like receptors (RLRs) and toll-like receptors (TLRs). Their engagement results in the activation of multiple transcription factors, including the nuclear factor kappa-light-chain-enhancer of activated B cells (NF-kB) and interferon regulatory factors (IRFs), that enhance interferons and various other inflammatory cytokines and chemokines orchestrating the innate and adaptive immune response. RNA viruses that replicates in the cytosol are recognized by RLRs RIG-I and melanoma-associated differentiation antigen 5 (MDA5), activating downstream IRF3 and NFKB to induce type I interferon production [21].

For ZIKV the infectious capacity seems to be primarily dependent by prM, a structural protein known as pre-membrane protein in ZIKV, and secondly by the ability to evade type I IFN responses, achieving long-term infection. It seems that the protein NS acts in modulating IFN signaling in favor of the viral immune escape. In summary, NS1 stabilizes caspase-1, NS2A down-regulates the promoter activity of IFN-β, NS3 protein blocks RIG-I and MDA5, while NS4A at the mitochondrial level and NS5 guarantees methyltransferase and RNA-dependent RNA polymerase activities [22].

Viral entry may occur also via the process of ADE of infection, where the quantity and the avidity of the antibodies produced, at the second viral encounter, are extremely important for tilting the table from protection to disease. In fact, antibodies generated form the primary infection that are sub-neutralizing in quality (low avidity) or quantity (low concentration) can bind a cross-reactive virion and, as immune complex, gains access to permissive cell bearing immunoglobulin Fc receptors (FcRs) such as FcRγ. This event can lead to increased viremia and viral burden in other tissues, occurring to increased disease severity for the host. On the contrary, when high avidity and concentration antibodies are produced the neutralizing response determines protection. Recently, a study showed ADE during ZIKV infection when a murine model lacking STAT2 passively received DENV-immune human sera [23]. Furthermore, few studies using several monoclonal antibodies have shown that all neutralizing antibodies can promote ADE in vitro when used at sub-neutralizing concentration [24,47,48]. Neutralization versus enhancement is just a function of the stoichiometry of antibody binding to the viral particles [49]. From the analysis of mAbs from ZIKA-infected humans and mice has emerged the crucial role of the E protein in protective immune responses. Different groups have studied highly neutralizing antibodies recognizing specific epitopes of this protein [50,51,52,53,54] and more than three classes of antibodies have been characterized. The first recognizes a quaternary epitope across the E protein (that cross-reacts with DENV) neutralizes ZIKV with high potency [55], protects murine models from ZIKV infection [56,57] or vertical transmission [56], and has therapeutic properties in non-human primates (NHP) [58]. A second class of protective antibodies binds the lateral ridge of DIII and blocks ZIKV infection preventing membrane fusion [59]. A third class recognizes an epitope among the E protein dimers and also protects against the transmission in mice [17,50]. A forth class of protective mAbs has been reported to bind additional sites within E-DI and E-DI [54], although analysis in pregnancy models are missing.

Ultimately, highly cross-reactive antibodies that target the fusion loop in E-DII were not efficiently neutralizing in vitro and showed no protective activity in vivo [52,57,60]. Induction of cross-reactive antibodies that recognize non-accessible epitopes may not be ideal for a vaccine or for passive immunization, as they may induce ADE. Some studies about DENV suggest that ADE versus negligible effects of antibodies may depend on engagement of different signaling cascades, as well as the engagement of competent/incompetent forms of FcγR1 and FcγRII, or via the downregulation of key antiviral molecules such as IRF-1 and signal transducer and activator of transcription 1 (STAT-1) and increase of specific cytokines such as tumor necrosis factor (TNF), interleukin-6 (IL-6) and IL-10 [61,62,63,64,65].

Although these have been studied for DENV, it does not take that far to assume that those mechanisms could be used by ZIKV. Systemic studies using mouse models of ZIKV infection have shown that anti-interferon-α/β receptor (IFNAR) monoclonal antibody-treated wild type animals, but also Ifnar1−/− knockout mice, as well as Stat2−/−, or Irf3−/−Irf5−/−Irf7−/− triple knockout mice, can die from ZIKV infections; while opposite was the effect upon the wild-type, Irf3−/−, Irf5−/−, Mavs−/−, Tmem173(STING)−/−, and Mb21d1 (cGAS)−/− mice [66,67,68,69,70]. Taken from evidences that show that Stat2−/− mice can succumb to infection with ZIKV [68], contrarily to DENV [71,72], the STAT2-mediated signaling may be seen as key player against ZIKV.

Furthermore, in addition to the claimed role of antibodies, it has been demonstrated the role of protecting cross-reactive CD8+ T cells seen in HLA transgenic mice, and as well suggested as tool for future vaccine development [12]. Additionally, passive transfer of CD8+ T cells from Zika-immune to naïve mice have revealed a relevant reduction of viral replication [73]. ZIKV infection during pregnancy induces a memory response, protecting from a secondary challenge, but not dependent on CD8+ T cells [74]. Along with those results, more studies are needed to fully characterize T cells that persist in CNS and understand whether they could contribute to inflammation, worsening the neurological disease [75].

Lately, a massive work has been conducted upon vaccine development. The emergency in the Americas and the severe congenital defects triggered the development of different vaccines that were advanced into clinical trials. Indeed, multiple candidates have shown promising results in both animal models and phase I clinical trials. Until 2019, phase I clinical trials with data on animal experimentation comprised mostly DNA or mRNA vaccines having prM/E proteins as immunogen. One of the DNA-based vaccine, named VRC5283, has showed relevant protection in NHP model, and demonstrated immunogenicity in humans, once evaluated safety and efficacy of a three-dose regimen. It has recently completed the phase II in December 2020 (Available Online: https://clinicaltrials.gov/, accessed on 31 August 2021, NCT03110770). All types of vaccines are well described elsewhere [5,76,77].

In summary, many of the current vaccines against ZIKV induce high levels of neutralizing antibody and antiviral CD4+ and CD8+ T cell activity. In human clinical trials, three doses of a DNA vaccine encoding for prM-E or two doses of inactivated virion (ZPIV) achieved mean titers above the 1/100 threshold leading to virus protection [78,79]. In the majority of trial participants, neutralizing antibody titers were induced, comparably to preclinical models. However, important challenges undermine the clinical development; in fact, with the current reduction in ZIKV transmission, a phase III clinical efficacy trial could prove challenging to execute.

Interestingly, it has recently been shown that tetracycline-inducible vaccinia expressing prM and E protein induced high anti-E IgG titers in vitro; on the other hand, in vivo, mice treated with anti-IFNAR1 and vaccinated with a single dose were protected from ZIKV, with an increase of interferon-gamma (IFN-γ)-secreting splenocytes and the induction of a humoral response [80].

## 5. Biological Modeling for ZIKV Research

Getting access to human or primate fetal brain samples is extremely difficult, thus in vitro models offer an essential alternative. Nowadays, traditional cell cultures and classical virology/microbiology techniques are still in use for exploring viral pathogenesis and carrying out antiviral research, but they have reached their dusk.

ZIKV can replicate within a large range of mammalian cell lines as thoroughly reported [81]. Studies on cell cultures and nonhuman primate and mouse models, as well as human clinical samples, suggest that ZIKV is pantropic. Because of its distinct complexity, it has been very difficult to model human brain development using animal systems [82]. So far, it is well-accepted that ZIKV affect neural progenitors, thus basically causing brain size loss.

As ZIKV infection prevented neurosphere formation, these in vitro models were very useful for discovering the special features of the virus, in contrast to other members of its family. As the neurospheres model highlighted in the past [83], the use of more advanced 3D engineered tissues may decisively help to unravel ZIKV prenatal infection, that has not been understood using the 2D “flat” cell model. In fact, brain organoids can link the gap between 2D cell cultures and the in vivo preclinical assays as they could recapitulate the entire viral life cycle. Human 3D cell-based models showed considerable capacity for studying virus-host interactions.

Organoids play an important role in mimicking a viral disease and researchers need to exploit humanized 3D models for a deeper understanding of human infections. Specific human biological phenomena are not amenable in animal models. In fact, the human brain is more complex than the murine one, in relation to specific resident cell populations, their abundance and metabolism. In fact, it has been hard to describe the effect of the virus during the neurodevelopment in animal models due to the species-specificity of the infection [84].

Organoids represent an improvement in human tissue modeling and accessibility to study many human organs and diseases. They are an extraordinary example of 3D cell culture that differ from simpler spheroid models (e.g., scaffold-free cellular aggregates) because they originate through the proliferation of progenitor cells that self-organize into clusters, which build up the architecture and resemble the functionality of the native tissue [85]. The cerebral organoid system exploits the properties of human induced pluripotent stem cells (hPSCs) to generate spontaneously neural tissues that form functional different brain regions. Original protocols, now inspiring the whole neuroscience community, were developed by Lancaster and Knoblich [86], less than 10 years ago.

Polarity and morphogens (also known as patterning factors) can determine, polarize and differentiate specific brain-regions. The production of specific forebrain, midbrain or hindbrain organoids has the advantage to be more consistent in terms of cell composition [87]. Additionally, direct approaches with morphogens can generate specific brain’s regions [88]. Despite the enormous technical advances in the last five years for generating organoid cultures, new advanced protocols are needed to fill the absence of the immune system components and the lack of specific cell’s phenotypes featured at different stages of brain development. However, brain organoids are a versatile experimental platform for modern virologists and immunologists to recapitulate ZIKV pathogenesis. According to the specific resembled tissue, viral infection can vary leading to local damage in regionally patterned organoids [38]. The reliability of human organoids in mimicking fetal brain development is in contrast to animal models which do not always reproduce the human physiology [89]. Microcephaly, one of the most severe complication, can be resembled by organoids thanks to their plasticity in changing morphology, size, polarity and tissue architecture. Furthermore, mimicking the biological barriers would be more reliable in 3D cultures as the tridimensional environment enables cells to expand in multiple directions, shaping diverse niches that enable cell lineage specification. An overview of the possible advantages provided by organoids [90,91], that can be used in flavivirus research, are summarized in Table 1.

## 6. Animal Models: Cons Outweigh the Pros?

Animal models have been employed to study the effects on the risk populations. While few studies tried to understand the viral effect during pregnancy, others focused on ZIKV infection on adults and neonates, and few others explored its sexual transmission, which is unique within the Flavivirus genus.

Whenever a new viral threat emerges, rodents are often the most common animal model taken in consideration but not necessarily a guarantee of a proper human surrogate. For instance, in the context of the last coronaviruses pandemics, SARS-CoV2 was shown to have poor affinity for the mouse homologue angiotensin-converting enzyme 2 (ACE2) that is strongly bound in humans [92].

In ZIKV field, mice models have enormously contributed to the current knowledge about the innate and the adaptive immune response, as well as the virus life cycle, tropism, transmission route, and possible treatments. Most mice models are generated based on the type I IFN pathway genes impairment making mice susceptible to the Flaviviridae family (as well as to other Arboviruses) infections. In the same way, the dexamethasone-induced immunosuppression is an alternative way to generate murine models for ZIKV research, providing human-like symptoms with multiorgan involvement [93]. Studies on different mice strains led to realize the relevant differences in symptoms severity between ZIKV African or Asian lineages, with the first being more inflammatory than the other, generating different immune profiles after the infection’s onset [94].

Additionally, neocortex formation is accounting for the highest human cognitive functions, but given the neocortex’s different developmental pattern and architecture in humans and rodents, the latter are not often appropriate for analyzing a specific time-frame of infection or neural progenitor’s phenotype. Another shortcoming in murine models is a low gyrification index [95], thus a differential expansion of the cerebral cortex that contribute to a certain discrepancy in ZIKV viral infection hallmarks. On the other hand, mice are generally easy to manage, with a short life cycle and gestational period, and a wide possibility of transgenic manipulation. Although microcephaly can be analyzed in murine models, the gestational period is too short in comparison to the human one, thus a pre-natal ZIKV infection model is missing. Conversely, pregnant primates transmit the virus [96] and exhibit fetus lesions similarly to those observed in humans with congenital Zika syndrome (CZS) [97].

NHPs have also been used as animal model for studying the virus life cycle and pathogenesis, as ZIKV also has them as hosts and reservoirs. The first animal model described susceptible to ZIKV was in fact rhesus monkey [98], with historical discovery in 1947. Among many, critical differences of the immune system among murine models and NHPs need to be taken in account as they complicate the potential transferability of the results. NHPs are considered an appropriate animal model to study vaccine-mediated protection and to understand the mechanisms of maternal–fetal ZIKV transmission [99]. Multiple vaccination against ZIKV tested in NHPs produced neutralizing antibodies and were protective against following challenge [100]; in these animals, while the presence of antibodies correlated with a rapid clearance of viremia, the virus persisted in lymph nodes [101].

Alternative models are *stat-2*-deficient guinea pig and hamsters, with certain range of advantages, however they cannot resemble many clinical aspects of ZIKV infection and they are relatively unexplored in therapeutic research, to the best of our knowledge [102,103].

Above all, it must be remembered that animal models carry their own microbiota, which composition is different from human’s, influencing the pathogen behavior, and adding interferences in clinical manifestation of the disease. In Table 2 is displayed a comparison of the main preclinical models suitable for Zika research.

## 7. Organoid-Based Studies

### 7.1. State of the Art

The first organoid model mimicking brain tissues derived from embryonic stem cells (ESCs) and was used to study ZIKA pathogen-host interaction. A link between MR766 ZIKV strain and TLR3 was identified both in human ESC organoid cerebral model and in murine neurospheres as well [104]. A second study using ESC-derived brain organoids showed how ZIKV infection changed DNA methylation of neural progenitors, cortical neurons and astrocytes, affecting genes correlated with neuropsychiatric disorders, thus with neurological ZIKV infection sequelae [105]. Cerebral organoids made from iPSCs were optimized for obtaining basal ganglionic eminences (GE)-like structures and inhibitory interneurons and validated compounds, such as duramycin, were capable of partially rescuing viral infection and mitigating ZIKV cytopathic effects [106]. In order to study the neurotropism of ZIKV, different level of complexity of cerebral organoids were developed, resembling the multilayer organization seen in the human CNS. Initial studies describing human iPSCs-derived brain organoids, whose results were published in 2016 by Cugola et al. [107], Garcez et al. [108] and Tang et al. [109], were fundamental to understand ZIKV targeting neural progenitors. Additionally, the research about potential inhibition of putative receptors responsible for virus entry [110] was supported by iPSC-organoid technology; for example AXL [111], was discovered as indispensable factor for infection and now considered a promoter of viral persistence thanks to organoids [112]. With the advent of the forebrain-specific organoids protocols and the results published by Qian et al. [113], Xu et al. [114] and Yoon et al. [115], both the proliferation interference upon brain progenitors and the specific pathogenic mechanisms on human brain development were understood at higher resolution grade [116].

Other types of cellular models were differently useful for ZIKV research. Putative ZIKV receptors (AXL and TYRO3) have been previously identified in 2D cell lines but were not confirmed as positive hits when organoids were used [38]. ZIKV E protein was proven responsible for disrupting neurospheres’ cells migration, impairing their growth, and promoting an early differentiation of neural stem cells [117]. Nonetheless, maternal transmission was supported by observations on an in vitro biomimetic placenta tissue [118]. Another in vitro surrogate, a cell-line based organotypic model of human syncytiotrophoblasts, helped studying maternal transmission. In particular, choriocarcinoma JEG-3 cells were added in co-cultures with beads of primary placental fibroblasts or endothelial cells [119].

The 3D systems can also simulate basic defense mechanisms. In fact, trophoblasts isolated from full-term placentas resist infection by diverse viruses, including ZIKV, and transfer this resistance to nonplacental cells through the activity of paracrine effectors, including the constitutive release of type III interferons (IFNs). It has been developed a 3D cell-line–based model of human syncytiotrophoblasts, cells that lie in direct contact with maternal blood, that recapitulate the antiviral properties of primary trophoblasts through the constitutive release of type III IFNs (IFNλ1 and IFNλ2) and become resistant to ZIKV infection [119]. A similar model was suitable for studying vertical transmission, with much interest in exploring the congenital disease pathogenesis [120].

### 7.2. Most Recent Findings

As already reported in 2018 by Majolo et al., the increased use of organoids and other relevant 3D cellular models has followed the clinical and research urgency. By modeling key functional characteristics of the pertinent tissue and allowing replication, organoids are currently an important experimental virology platform [121]. The most recent study showing the superiority of brain organoids in modeling ZIKV infections has uncovered virus-specific mechanisms by comparing ZIKV with another common virus causing newborn morbidity, such as herpes simple virus (HSV). Both viruses were confirmed infecting human neuroprogenitor cells, which represent the main target. Briefly, ZIKV infection reduced lumen size, while HSV-1 resulted in a greater general damage and enlarged lumens. Both the viruses attenuated IFN-I responses in organoids stronger than 2D cultures. Significantly, the attenuated interferon response described in organoids is consistent with the low immune reactions reported in the microcephalic ZIKV-infected patients. Additionally, such research has shown that these viruses differentially engage the interferon system while organoid defects can be rescued by specific type I interferon treatment. In particular, Krenn et al. [122] highlighted the efficacy of IFN-β in ameliorating organoid phenotype infected by ZIKV.

Cerebral organoids obtained from iPSCs were used to evaluate a new class of antiviral compounds. The most promising compound, an 8-Oxoguanine DNA Glycosylase (OGG1) inhibitor, interacts with members of the Hsp70 chaperone network, disturbing ZIKV particle release and rescuing ZIKV-induced toxicity [123]. Thanks to organoids, it has been shown that this class of small inhibitors can vary the late steps of the virus life cycle. The identification by mass spectrometry of ZIKV peptides, that are processed by T cells, were shown to recall memory T-cell response, thus they can be used as epitopes in experimental vaccine formulations [124]. Furthermore, enoxacin antibiotic treatment supports a RNA-interference in mammalian host, in particular preventing ZIKV infection of NPCs with loss of proliferation and shrinking in brain organoids [125]. Not last, methylene blue (MB), a water-soluble drug historically used for malaria, has recently found to inhibit NS2B-NS3 proteins activity. Intriguingly, this drug already tested as antiviral, was effective at both entry and post infection stages and protected mini-brain organoids from ZIKV infection [81]; those sliced samples revealed no virions production after treatment with MB at 1.5 μM. The authors also showed a reduced viral viremia in their murine model.

While a pronounced infection of microglia may be expected, astrocytes are more mysterious and recently focus of further investigations. Among human iPSC-derived cell types, in fact, astrocytes were found preferentially targeted by ZIKV in comparison to neurons, and more prone to oxidative stress, mitochondrial and DNA damage after ZIKV infection [126].

Organelles can be utilized by pathogens and ZIKV specifically reorganizes the endoplasmic reticulum. In particular, ZIKV-infected brain organoids indicated transformation of ER sheets to convoluted membranes, visible by super resolution microscopy [127]. In order to avoid sacrificing spatial information in brain organoids, a fluorescent microscopy platform associated to an automated multiscale comparative analysis (clearing, labeling and imaging) of intact processed organoids has been proposed. The developed SCOUT pipeline for multiscale hyperdimensional analysis of organoids works on 3D resolution image datasets to detect and section single cells. This will enable the examination of the spatial reorganization of cells and tissue during organoid maturation and post-infection. By this technology, it was possible to quantify the impact of ZIKV infection on brain development and analyze rare cell population, such as TRB1+ cells, during cytoarchitectural changes [128].

### 7.3. Current Limitations

All animal models show a certain grade of disparity between gene and protein expression compared to humans, making likely unsuccessful unbiased studies. As for primary human neurons and other cell tissues used to modelling ZIKV infection, ethical permissions are always required and represent a limitation for assembling organoids, which are made of iPSCs or derived from primary biopsies. Organoids derived from brain, an organ that is mainly constituted of non-epithelial cells, requires sophisticated strategies [129]; those available include two big groups, namely cerebral organoids and brain region-specific organoids [130,131].

The majority of current brain organoids models ZIKV infection lacking the immune and vascular systems [130]. Specifically, the vasculature is important for a proper modelling of fetal brain hypoxia considering that without blood vessels, a proper nutrition cannot be given to all the cells which the brain is made. In fact, brain organoids, implanted into the adult mouse brain cortex, got vascularized demonstrating an improved neuronal activity without necrosis [132]. Up to date, new models are developing to supply organoid artificial micro-vascularization, such as cerebral cortex organoids [133].

Cerebral organoids have cell types similar to those found in the human brain but those are not organized in the same way. The gyrification of the human neocortex has not been implemented in most organoid protocols, but recently an effort has been done for this purpose [134], concomitantly with the engineering of flat brain organoids [135]. As weakness, non-patterned self-organizing cerebral organoids can obviously display low reproducibility because of the different source of iPSCs and an arbitrary positioning of developing of brain regions. Moreover, the glia compartment is critical for obtaining relevant results and correctly addressing all ZIKV-induced cell changes after infection.

To note, immune cells are derived from yolk sac and mesoderm while brain organoids are usually derived from neuroectoderm, thus the microglia population is difficult to be displayed (e.g., differentiated in organoids) or included in the model [136]; of course some studies have raised doubts about this limitation [137] and suggested to provide a better representation of the nervous system environment. Human organoids missing immune cells are likely to fail at examining the viral mechanism of entry in the CNS. For this reason, it has been proposed the use of human post-mortem microglia-containing organoids for modeling ZIKV immune reaction, however this application is still in development.

Another aspect to consider is gender-related differences for ZIKV infection. It seems that a strong response inhibits ZIKV to control the infection in females, while in males, the infection correlates with viral persistence [138]. In line with it, a gender-specific immune system may play differently during sexually transmitted infection pathogenesis, thus the request of immunocompetent new sexual transmission model relying on organoids and influenced by hormones are as well demanded. A promising strategy for receptors discovery and viral tropism may be modeling virus latency, such as it seems relevant in male reproductive tract and glial cells. Unfortunately, even though the brain organoids are viable for weeks, it is unfeasible to maintain such very long-term cultures. 

Last, organoid protocols still lack standardization and the production of organoids is relatively expensive. The complexity associated to brain organoids brings challenges regarding their 3D morphological, molecular and neurophysiological analysis [87]. Being at the interface between in vivo and in vitro, advantages as well as disadvantages from both systems will be translated to organoid application.

## 8. Future Advances in ZIKV Research

### 8.1. About Organoids

It is known that ZIKV is able to cross the blood–brain barrier (BBB), reaching the CNS without damaging it [139]. Briefly, it seems that the microglia cells are the reservoir responsible for crossing the placenta while macrophage populations are responsible for BBB penetration. Understanding how ZIKV passes through the placenta or BBB may help fighting other viruses as well. In general, advanced 3D placental barrier models [140] can be based either on 3D bioprinted biological membrane or co-culture systems [141].

In addition to vaginal organoids [142], the development of organoids resembling the sexual reservoirs [143,144,145], representing both female and male [146,147,148] reproductive tracts, such as cervical [149,150] and testes organoids [112,151], may help to unravel sexual transmission and pathogenesis, especially the male-to-female route which is enhanced by asymptomatic infected males [152].

Organoids can be included in organ-on-chips [153,154], as this technology may soon give an advantage in circulation of the delivered pathogen and provide a dynamic system, with adequate oxygen and nutrients renewal and cellular waste removals from the organoids [155]. With the advantage of microfluidic devices, the delivery in a temporally and spatially regulated manner of morphogens during the organoid formation may be optimized, while antiviral compounds upon infection and therapeutics after that can be tested in dynamic conditions with cell model perfusion.

Gyrification of the human brain is connected to the compressive mechanical forces and microfluidics can be useful trying to achieve this particular process. Additionally, bioengineering and bioprinting technology may facilitate the control of spatial and temporal distribution of the organoid cellular system and implement extra cellular-matrix or vascularization [156] usually lacked by this model. Current vascularization approach includes both the introduction of microfluidic devices and the transplantation of organoids into immunodeficient animals [157]. Importantly, integration of microfluidics would constitute a possible advancement for establishing perfused vasculature into organoids [158].

By using a personalized medicine approach, genome-wide screens, aiming to find host factors which restrict the viral disease or promote viral replication, would be progressively more applied to organoid cultures [38]. Next generation sequencing (NGS) is currently employed to identify virus variants and monitor widespread transcriptional changes upon infection, but gene expression levels do not necessarily reflect proteins abundance. On the other hand, proteomics techniques have higher sensitivity and detect post-translational modifications (PTMs) as well. They can map virus−host protein interactions and possible PTM modifications upon ZIKV infection just using organoids [159]. Knowing host-pathogens protein interactions would be fundamental when a new pathogen causes an epidemy [160]. Nonetheless, proteomic analysis on organoids infected by ZIKV has not been yet reported.

Lately, Wang et al. used patient’s cells to generate asparaginyl-tRNA synthetase1 (NARS1)-mutated cortical brain organoids for modeling microcephaly, showing reduced proliferation of radial glial cells and demonstrating the importance of protein translation for brain development [161].

In addition, to overcome missing interregional interactions by region-specific organoids, the researchers are developing (for various organs) assembloids, mainly formed by joining patterned pre-mature or mature region-specific organoids [162]. For instance, fusion of cortical brain organoids and striatal brain organoids promoted neural projections extending unidirectionally from the cortex into the striatum, as in vivo.

All these advances may soon be applied to ZIKV research to generate sophisticated datasets and relevant knowledge about the viral pathogenesis and immune evasion.

### 8.2. About Therapeutics

Given the urgent need for ZIKV prophylaxis and treatment, repurposing of approved drugs appeared to be a viable and immediate solution. In fact, already in 2016, chloroquine treatment was shown to partially rescue mouse neurospheres and to protect fetal mice from microcephaly [163]. Many other anti-malaric compounds such as chloroquinones [164] were thought valuable antivirals and repurposed for ZIKV treatment, such as mefloquine [165].

To date, no therapeutic treatment is available to treat ZIKV-infected patients and research in therapeutics has focused on many natural compounds for years. Hydroxycholesterol was the first potential antiviral blocking ZIKV entry, attenuating ZIKV-viremia in non-human primates, microcephaly in mice and seen preventing the infection in human cortical organoids [166]. Another one, hippeastrine hydrobromide (HH) and amodiaquine dihydrochloride dihydrate (AQ) showed the potency of eliminating the virus in human pluripotent stem cell-derived cortical neural progenitor cells (hNPCs) [116].

Other natural compounds have currently provided encouraging results. For example, cavinafungin, a natural compound [167], targets the endoplasmic-reticulum signal viral peptidase impairing the replicative cycle of flaviviruses; labyrinthopeptin, a lantibiotic synthesized from bacteria, has shown in vitro potential of virolysis by perforating lipid membranes [168]; another one, emetin, an anti-protozoal agent, inhibited viral replication and viral entry of ZIKV and Ebola virus (EBOV) [169]. Curcumin interfered with ZIKV binding to cells [170] and is proposed as diet-derived therapeutics as proven to have antiviral effects in Vero cells at concentrations ranging from 12.5 to 50 μM [171]. Additionally, in Vero cells, marine diterpene dolastane showed virucidal activity against ZIKV [172]. Epigallocatechin gallate (EGCG), the most abundant catechin in tea, inhibited ZIKV entry on host cells independently from the blockage of receptors [173], with a mechanism inhibiting the viral NS3 helicase [174]. Interestingly, ginkgolic acids (GA) are alkylphenol constituents of the leaves and fruits of *Ginkgo biloba* that have raised considerable interest due to inhibiting human cytomegalovirus (HCMV) genome replication and ZIKV infection of normal human astrocytes (NHA) [175]. Additionally, resveratrol, with common pleiotropic healthy effects, has shown anti-ZIKV activities by reducing its titers and mRNA copy number [176]. On the other hand, host restriction factors such as viperin, which is an interferon-inducible protein, should be characterized in more relevant physiological system as well [177].

Clearly, all the mentioned compounds need to benefit from brain organoid in vitro analyses to be entitled as truly effective against ZIKV.

## 9. Conclusions

ZIKV infection constitutes a global harmful threat. The current COVID-19 pandemic has shown that new tools and technologies for the advancement of basic science are required and the organoid platform could participate as pre-clinical platforms for studying all pathogens. In virology, in vivo models are usually made up by specific gene manipulation or applying harmful conditions to animals, while recent advanced in vitro models, definitely cruelty-free, can be directly generated without prior knowledge of the specific responsible genes, obtaining clinical specimen from the patients [82]. Considering the huge and sudden increase of publications about organoids as effective in vitro models for unraveling host-viral interactions, we found it useful to review the most recent available evidences and offer key points for shaping future directions on ZIKV research. Surely, human organoids have shown the potential to resemble functional and physiological responses to ZIKV infection; many researchers have demonstrated that they can be a useful tool for pre-clinical investigations and therapeutics studies. New research areas, not yet addressed or that are still at their beginning, are encouraged and will powerfully expand the knowledge on this pandemic-prone pathogen.

Proper integration of disease control measures and effective surveillance programs should be supported by the continuous improvements both in diagnostic methods and research models, such organoids, to guarantee the emerged “one-health” point of view [178].

In conclusions, the application of organoids represents a relevant approach for deciphering pathogenicity mechanisms in humans and evolving valuable predictive models. Human organoids uniquely help researchers in the world collective fight against Zika disease [9].

## Figures and Tables

**Table 1:** Advantages provided by organoid cultures in clinical virology research.

Organoid Application	Possible Advantages
biomarker discovery	Identification of specie-specific markers, restricted to the organ development stages
pre-clinical toxicity	stem cell contribution and regeneration properties during infection
co-infections	viral competition for cellular and subcellular targets
molecular tools and gene editing	changes affecting physiology of surrounding cell types
antiviral discovery	analysis of the viral entry inhibition and the stop of replication
monoclonal antibodies administration	assessment of physical biological barrier and efficient drug penetration
multiple vaccine scheme	testing vaccines or different vaccine schedules
clinical-derived samples	genetic viral susceptibility and personalized medicine

**Table 2:** Comparison between preclinical models suitable for Zika research.

Preclinical Model	Physiological Complexity	Translational Grade [91]	Current Advantages
Neuronal Cell Culture	Low	Medium	Low cost High throughput Highly accessible Easly infected
Brain Organoids	Medium	Medium *(ongoing evaluation)*	3D environment Developmental stages Organotypic cell presence Barriers between tissues
Animal Models	High	Low *(due to differences in metabolism)*	Sexual transmission Full brain features Systemic response Barriers between organs
